# Optimising Sleep Stage Detection Using a Minimal Non‐EEG Physiological Signal Set and Deep Learning

**DOI:** 10.1111/jsr.70266

**Published:** 2025-12-14

**Authors:** Ángel Serrano Alarcón, Maksym Gaiduk, Natividad Martínez Madrid, Juan Antonio Ortega, Ralf Seepold

**Affiliations:** ^1^ IoT Laboratory Reutlingen University Reutlingen Baden‐Württemberg Germany; ^2^ Lenguajes y Sistemas Informáticos Universidad de Sevilla Sevilla Spain; ^3^ Ubiquitous Computing Laboratory HTWG Konstanz Konstanz Baden‐Württemberg Germany

**Keywords:** deep learning, physiological signals, signal processing, sleep stages, U‐net

## Abstract

Automatic sleep stage classification is essential for enabling non‐invasive, at‐home monitoring. However, current methods often rely on electroencephalogram (EEG) signals and ad‐hoc development approaches that limit reproducibility. We present a reproducible engineering framework for a deep learning model based on the U‐Net architecture that classifies sleep into five stages (Wake, N1, N2, N3 and REM) or four (Wake, Light Sleep, Deep Sleep and REM) using only three easily acquired physiological signals: oxygen saturation (SpO), heart rate (HR) and abdominal respiratory effort (AbdRes). In contrast to most previous studies, our model provides sleep stage predictions on a per‐second basis, thus overcoming the limitations associated with fixed 30‐s epochs. The model was trained on the Sleep Heart Health Study—Visit 2 (SHHS2) dataset and externally validated on the Multi‐Ethnic Study of Atherosclerosis (MESA). Optimisation of the model was achieved via Keras Tuner with the Hyperband algorithm. The study achieved weighted F1‐scores of 68% (five‐stage) and 71% (four‐stage) with Cohen's Kappa of 0.61 and 0.67 on SHHS2, with consistent performance on MESA. These results demonstrate strong generalisation and suggest that this lightweight, EEG‐free approach offers a practical path towards scalable, clinically relevant sleep monitoring.

## 1 Introduction

When developing new ways to detect sleep stages, the scientific literature reveals some solutions that aim to use several approaches. These include the detection of sleep stages by using a single electroencephalogram (EEG) channel or the use of one physiological signal, such as heart rate (HR) (Mitsukura et al. 2020; de Gans et al. 2024). In addition, many of these works use a large set of signals or EEG signals, which facilitates the detection of sleep phases or even other sleep disorders (Imtiaz 2021; Gaiduk et al. 2023; Zhang et al. 2021). For example, in Huttunen et al. (2023), oxygen saturation (SpO_2_) is used in combination with other signals such as photoplethysmogram (PPG), nasal pressure, EEG, oronasal thermocouple and respiratory effort. This work uses a U‐Net model to classify five sleep stages, achieving an F1 between 68% and 79%, which is better when the number of signals used increases.

In addition, alternative sets of signals can be used, such as respiratory and movement signals (Stuburić et al. 2020). Furthermore, the use of portable monitors or the development of artificial intelligence (AI) models is also noteworthy (Choo et al. 2023). For instance, a minimal set of signals consisting of SpO_2_, HR, abdominal (AbdRes) and thoracic (ThorRes) respiratory effort has already been shown to be effective in classifying obstructive sleep apnea events (Alarcón et al. 2023). Based on the information extracted from the scientific literature in the field, using these signals separately or in combination with another set of signals to detect sleep stages through automatic methods yields promising results in the classification of five and four sleep stages (Gaiduk et al. 2018). Regarding HR, it was also used in conjunction with respiratory signals, yielding a Cohen's Kappa of between 0.50 and 0.55 for the categorisation of four stages of sleep (Choi et al. 2020; Sun et al. 2020). Furthermore, the use of AbdRes was shown to be effective in the assessing sleep stages, as evidenced by Hanna and Flöel (2023), where a deep learning model integrating AbdRes and the electrocardiogram achieved a Cohen's Kappa of 0.58 for the recognition of five sleep stages.

One of the most popular research areas is the development of AI models for automatic detection of a small set of signals. However, a standardised approach to developing a deep learning model for sleep phase detection, using a minimal set of non‐invasive physiological signals for the patient, and generating an automated prediction for clinical use has yet to emerge. Consequently, current alternatives to polysomnography (PSG), in conjunction with the application of AI, represent the majority of scientific publications in this field. For example, the open‐source Greifswald Sleep Stage Classifier, which includes only electrooculography (EOG) data, has been developed for the assessment of sleep stages (Haghayegh, Khoshnevis, Smolensky, and Diller 2020). The use of AI in conjunction with wrist actigraphy for the evaluation of sleep patterns has also been explored (Haghayegh, Khoshnevis, Smolensky, Diller, and Castriotta 2020). Furthermore, the Haghayegh Algorithm, which uses a combination of motion and HR variability data, has been used to develop AI models (Kim et al. 2022). Moreover, the application of deep learning to establish a clinical decision support system has been investigated (Jaqua et al. 2023). In the context of the development of AI models for the detection of sleep stages, a review of the scientific literature reveals that most scientific articles present developments in AI algorithms that achieve good results in the detection of sleep phases after trial and error without following a specific development methodology (Gaiduk et al. 2023). Although the results of these algorithms appear promising, the approach used for their development makes it very difficult to reproduce the results. It is not feasible in clinical practice because the development of algorithms is presented as an art rather than an engineering process. This fact means that although there are more opportunities to obtain more accurate results due to the larger number of signals used, the computational and data preprocessing costs are higher. Despite the accuracy that can be achieved with electroencephalography, the setup and signal acquisition process make it suboptimal for use as an alternative to PSG outside the laboratory. Additionally, using a portable monitor that is as uncomfortable for the patient as PSG would be impractical. Therefore, the ideal is to find a minimal set of signals that a portable monitor can conveniently collect, providing accurate results to detect different sleep disorders and not just sleep phase detection.

Sleep disorders significantly affect people's health (Mc Carthy 2021). Poor sleep quality causes fatigue the next day, but also sleep disorders can lead to different pathologies such as depression, heart failure, diabetes or dementia (Gaiduk et al. 2021). Therefore, it is crucial to assess sleep quality to make a diagnosis and act accordingly. Detecting sleep phases is essential to treat potential sleep disorders; however, this process requires specialised settings and clinicians; the sleep test often has long waiting lists, and the set of sensors used is cumbersome to the patient (Perslev et al. 2019). Although PSG provides highly accurate results, recent research focusses on reducing costs, minimising clinical resources and improving patient comfort while maintaining acceptable accuracy in sleep stage classification. Research on how to enhance this process has been ongoing for some time (Imtiaz 2021). In this scientific work, we propose the development of a deep learning algorithm for the detection of five and four stages of sleep (Wake, N1, N2, N3 and REM or Wake, Light Sleep, Deep Sleep and REM) following an engineering approach using Keras Tuner (Lamberta 2017). In addition, a small set of three physiological signals can be easily acquired without invasive methods for the patient. The classification was performed for five and four sleep phases, due to the difficulty in fully detecting five sleep phases with a high level of accuracy. This allowed the investigation of whether the grouping of N1 and N2 in light sleep and leaving N3 in deep sleep yielded superior results or, conversely, whether no discernible differences were observed. This engineering approach aims to establish a methodology to develop deep learning models for the detection of the stage of sleep. This can be achieved by focussing on relevant aspects such as the application of minimum preprocessing of the signals before training, the avoidance of a trial‐and‐error process, and the search for computational efficiency of the training without neglecting the best performance of the models. It is important to note that this fact is generally not considered when developing deep learning models for detecting sleep phases. This is particularly relevant in scenarios with limited access to high‐performance computing environments. Consequently, it is imperative to consider this aspect, as it facilitates the acquisition of optimal outcomes from AI models in terms of classification, whilst substantially minimising the computational demands of training and thus reducing the footprint. The model used is based on a U‐Net architecture, which has already demonstrated promising results in the field of sleep analysis (Perslev et al. 2019; Perslev et al. 2021). The signals used were SpO_2_, HR and AbdRes. In this paper, we will present the process followed during training, evaluation of the models, as well as the results obtained. Consequently, the three signals employed in this study, SpO_2_, HR and AbdRes, were selected to enhance the classification of sleep stages and facilitate their acquisition through minimally invasive procedures for the patient. Moreover, such a set of signals can be used to detect other sleep disorders (Haghayegh, Khoshnevis, Smolensky, Diller, and Castriotta 2020).

## 2 Methodology

**FIGURE 1:**
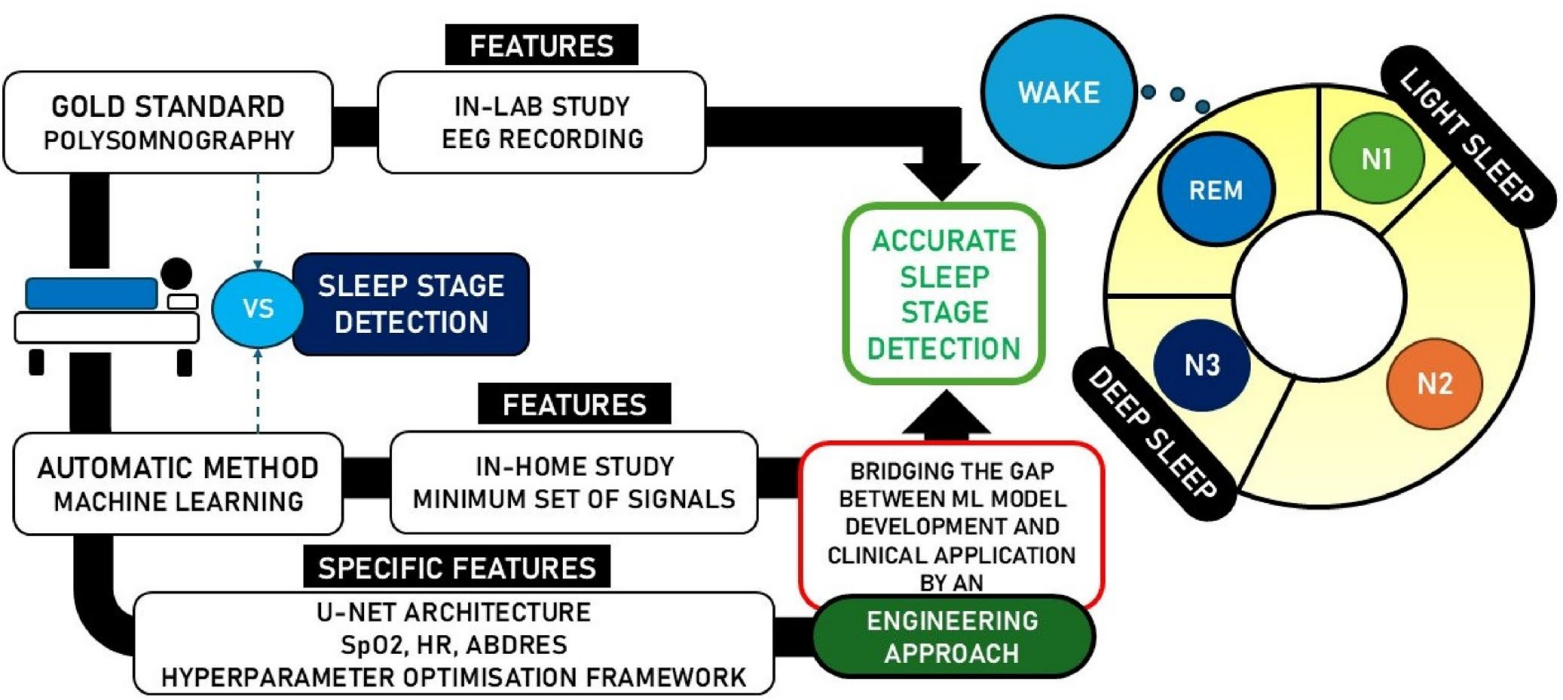
Workflow with the methodology applied in this scientific work. This is a comparison of sleep stage detection using standard polysomnography (PSG) versus an automatic machine learning method. The automatic method uses a minimal set of signals (SpO_2_, HR and AbdRes) and the U‐Net architecture to optimise hyperparameters.

This section presents details regarding the methodology used to develop AI algorithms. As previously stated in this scientific work, our aim is to adopt an engineering approach when developing AI models for detecting sleep stages. To achieve this, it is necessary first to select a suitable dataset comprising data that contains the information required and provides a sufficient quantity and quality of the data. The next step is to select the deep learning model. Following an extensive literature review, the optimal model architecture should be identified to achieve the best results with respect to the specific problem under consideration. Finally, training and optimal hyperparameter search are essential. Following the model training, the evaluation of the models will be carried out. Therefore, this section presents details about the dataset used, such as the size and content of the training, evaluation and test datasets. Specifics are also given about the preprocessing of the signals as a prior step to training the models. In addition, information about the model architecture and the stages of model training are discussed throughout this section. The workflow of the methodology employed is illustrated in Figure 1.

### 2.1 Data Selection

Data from the Sleep Heart Health Study (SHHS) and Multi‐Ethnic Study of Atherosclerosis (MESA) were used to develop and validate deep learning models. The SHHS can be found in the National Sleep Research Resource (NSRR) and is a multicentre cohort study implemented by the National Heart Lung and Blood Institute (NHLBI) to determine the cardiovascular and other consequences of sleep‐disordered breathing (Zhang et al. 2018; Howard et al. 1997). Two datasets were generated from this study: SHHS Visit 1 (SHHS1) and SHHS Visit 2 (SHHS2), which were recorded at different periods. A literature review did not reveal a definitive conclusion on the superiority of the SHHS1 or SHHS2 datasets when using deep learning models. Consequently, the decision was taken to employ SHHS2 for model training, given that software and hardware enhancements had been implemented to collect data, which can indicate enhanced data quality.

MESA is a six‐centre collaborative longitudinal investigation sponsored by NHLBI of factors associated with the development of subclinical cardiovascular disease and the progression from subclinical to clinical cardiovascular disease in 6814 black, white, Hispanic and Chinese‐American men and women aged 45–84 years at baseline in 2000–2002 (Chen et al. 2015).

The present study was designed with the specific objective of detecting sleep phases automatically from signals with durations comparable to PSG (i.e., ≥ 7–8 h), while minimising the necessity for preprocessing. In order to ensure data reliability and consistency, specific exclusion criteria were applied to the SHHS2 and MESA cohorts. Subjects were excluded from the study if annotations showed issues with sleep stage scoring, such as unclear stages or missing sleep phases, such as REM. For the subjects included in the study, physiological signals (SpO_2_, HR and AbdRes) were extracted, with the sampling frequency reduced to 1 Hz as required (SpO_2_ and HR at 1 Hz; abdominal respiration originally at 10 Hz). It should be noted that the present study exclusively utilised records containing a minimum of 10 h (36,000 s) of valid data, with the sleep stage labels being aligned accordingly. Consequently, due to hardware constraints, the signal duration was reduced to 28,000 s, yet 8 h of data remained for sleep phase classification. A comprehensive explanation of these details can be found in Section 2.2.

SHHS2 data was previously used to train AI models for sleep stage detection, with successful results (Hanna and Flöel 2023). Consequently, in the present work, SHHS2 was utilised for training purposes, while MESA was used as a non‐training dataset to investigate the generalisation of the models to new data. SHHS2 was used for both model training and testing. MESA was only used for testing. More details on the characteristics of the patients in the SHHS2 and MESA datasets for model training and evaluation can be found in Table 1. This table summarises the demographic distribution (age, sex and race), body mass index (BMI) and sleep‐related metrics (total sleep time and time spent in each sleep stage) for participants in the SHHS2 and MESA datasets. Values are presented as mean ± standard deviation. These characteristics provide context for training and evaluating the sleep stage classification models.

**TABLE 1:** Characteristics of the patients in the SHHS2 and MESA datasets.

Dataset	Number of subjects	Age	BMI	Gender	Race	TST (min)	SST (min)
SHHS2	855	66 ± 10	28 ± 5	Male: 365 Female: 485	White: 740 Black: 56 Other: 59	384 ± 68	Wake: 216 ± 67 REM: 81 ± 30 N1: 21 ± 13 N2: 220 ± 5 N3: 60 ± 40
MESA	931	69 ± 9	28 ± 5	Male: 412 Female: 519	White: 442 Black: 269 Asian: 121 Hispanic: 99	366 ± 78	Wake: 234 ± 78 REM: 65 ± 30 N1: 51 ± 32 N2: 210 ± 58 N3: 40 ± 35

*Note:* Body max index (BMI), sleep stage time (SST) and total sleep time (TST) are shown as mean ± standard deviation.

### 2.2 Data Pre‐Processing

One of the crucial aspects of this scientific work is to evaluate the model's behaviour using data, applying minor pre‐processing (avoidance of complex filtering techniques or feature engineering) before training the models. Signals that fed the models had a frequency of 1 Hz. On the one hand, SpO_2_ and HR were initially sampled at that frequency, while AbdRes was sampled at 10 Hz and down‐sampled at 1 Hz through an interpolation operation. The signals have a duration of 8 h. The set of signals was standardised (*z*‐score) before starting the search process for the best model.

Although a minimal preprocessing strategy was adopted to maintain the simplicity of the system and its applicability in clinical or home environments, it is important to clarify that advanced artefact removal techniques—such as high‐ or low‐pass filtering, principal component decomposition or multivariate outlier detection—were not applied. Other potential artefacts such as brief disconnections, motion‐induced noise or abrupt fluctuations within physiological ranges were not specifically treated. It was assumed that, by working with low‐frequency signals (1 Hz) and applying standardised normalisation (*z*‐score), the U‐Net model could implicitly learn to tolerate such variations. This represents a deliberate trade‐off between computational complexity and model robustness, and future work should evaluate the impact of unaddressed artefacts on the model's generalisation capability. Taking into account the hardware available for the development of this experiment, it was determined that the initial size of the signals in the dataset, which was 36,000 s, would be reduced to 28,000 s. This reduction was deemed necessary to facilitate model training in terms of computational efficiency.

#### 2.2.1 SHHS

**FIGURE 2:**
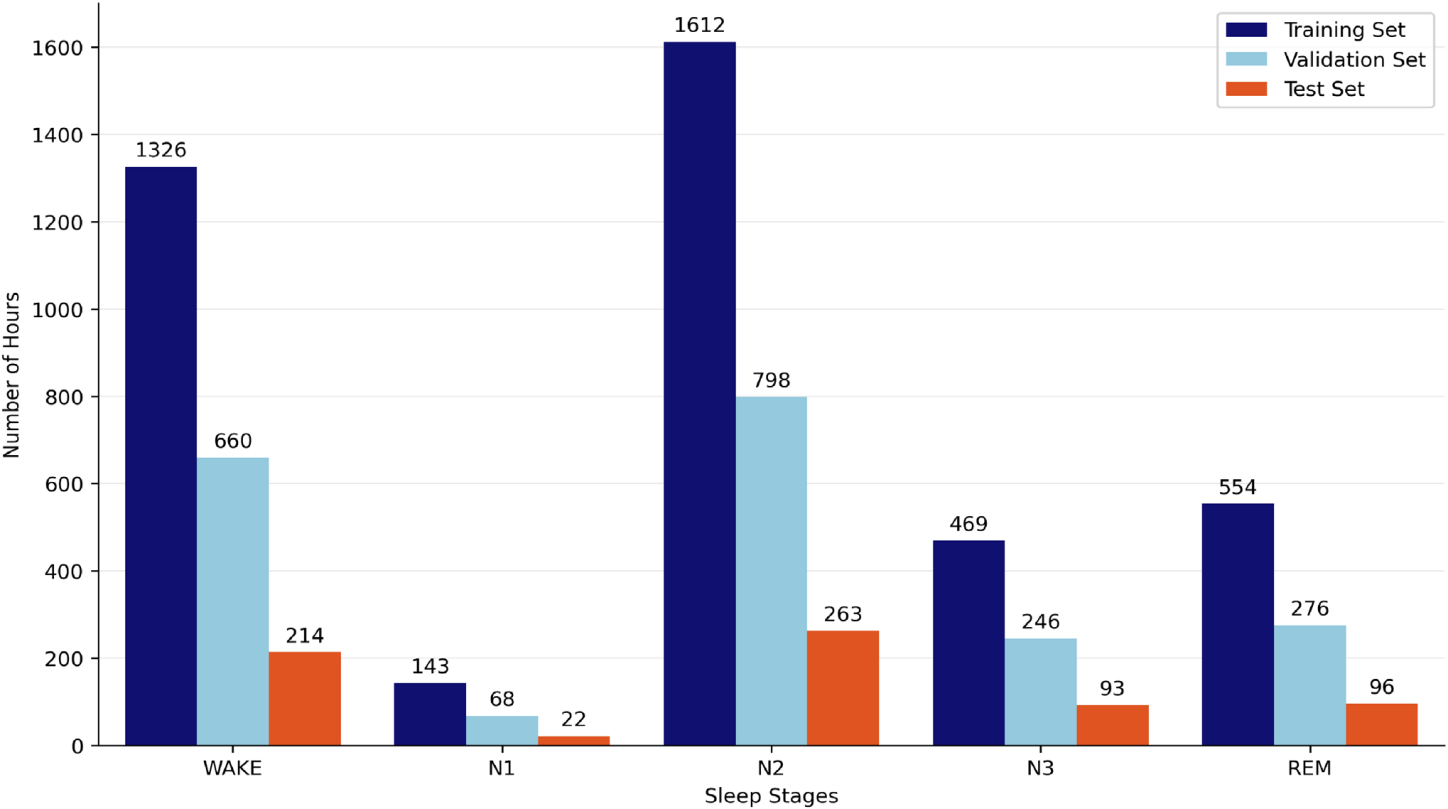
Distribution of training, validation and test sets of the model in the five‐stage (WAKE, N1, N2, N3 and REM) sleep recognition from the SHHS2 dataset.

After data preprocessing, only 855 SHHS2 patients met the criteria outlined in the previous section. The total dataset was then divided into three subsets: a training set comprising 513 patients (60%), a validation set comprising 256 patients (30%), and a test set comprising 86 patients (10%). Figure 2 illustrates the distribution of sleep phases across the five stages of sleep for each of the training, validation and test datasets.

**FIGURE 3:**
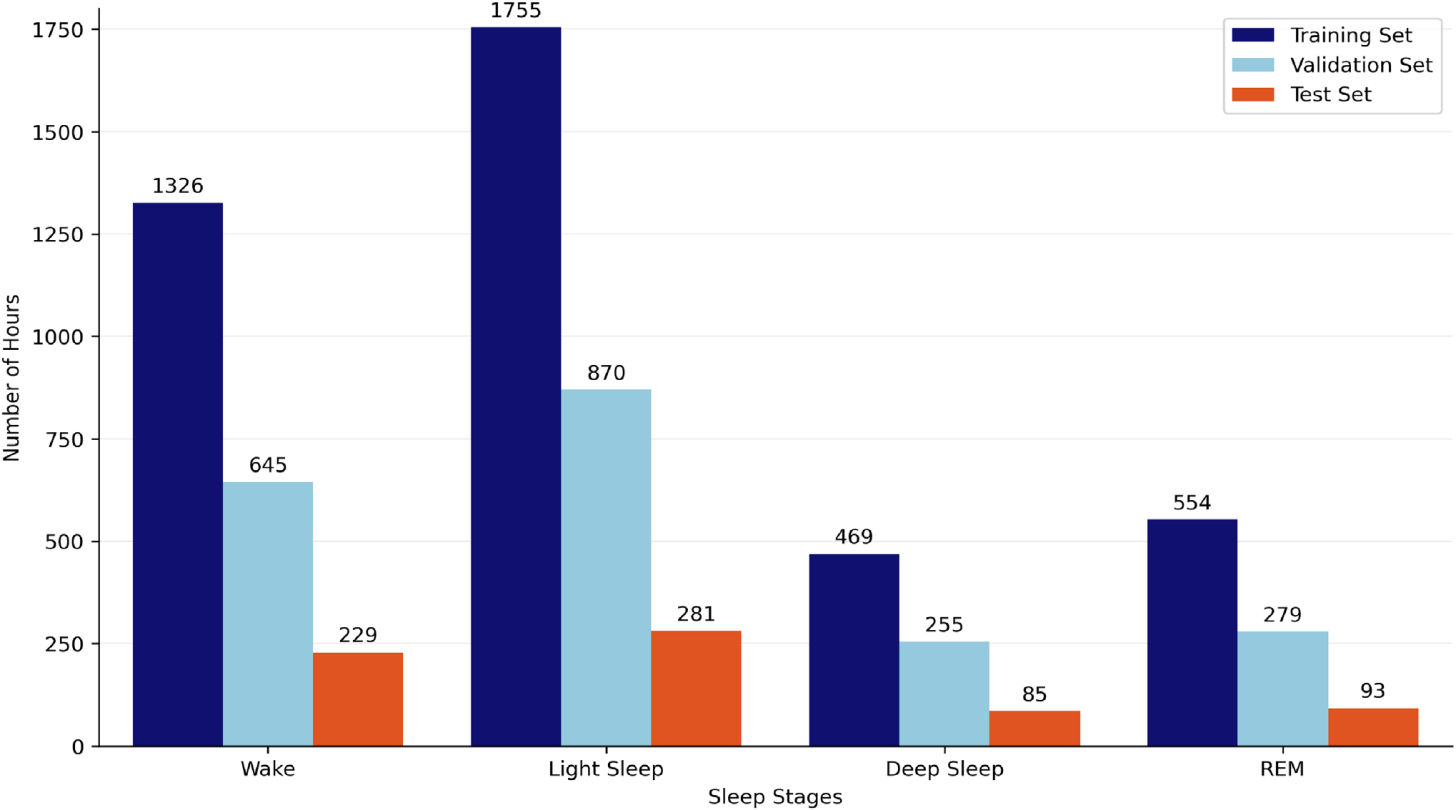
Distribution of training, validation and test sets of the model in the four‐stage (Wake, Light Sleep, Deep Sleep and REM) sleep recognition from the SHHS2 dataset.

On the other hand, for the classification of four sleep phases, the data distribution across training, validation and test sets can be seen in Figure 3. The smaller amount of data from N1 is balanced in this way through the merge between N1 and N2 into Light Sleep.

#### 2.2.2 Mesa

**FIGURE 4:**
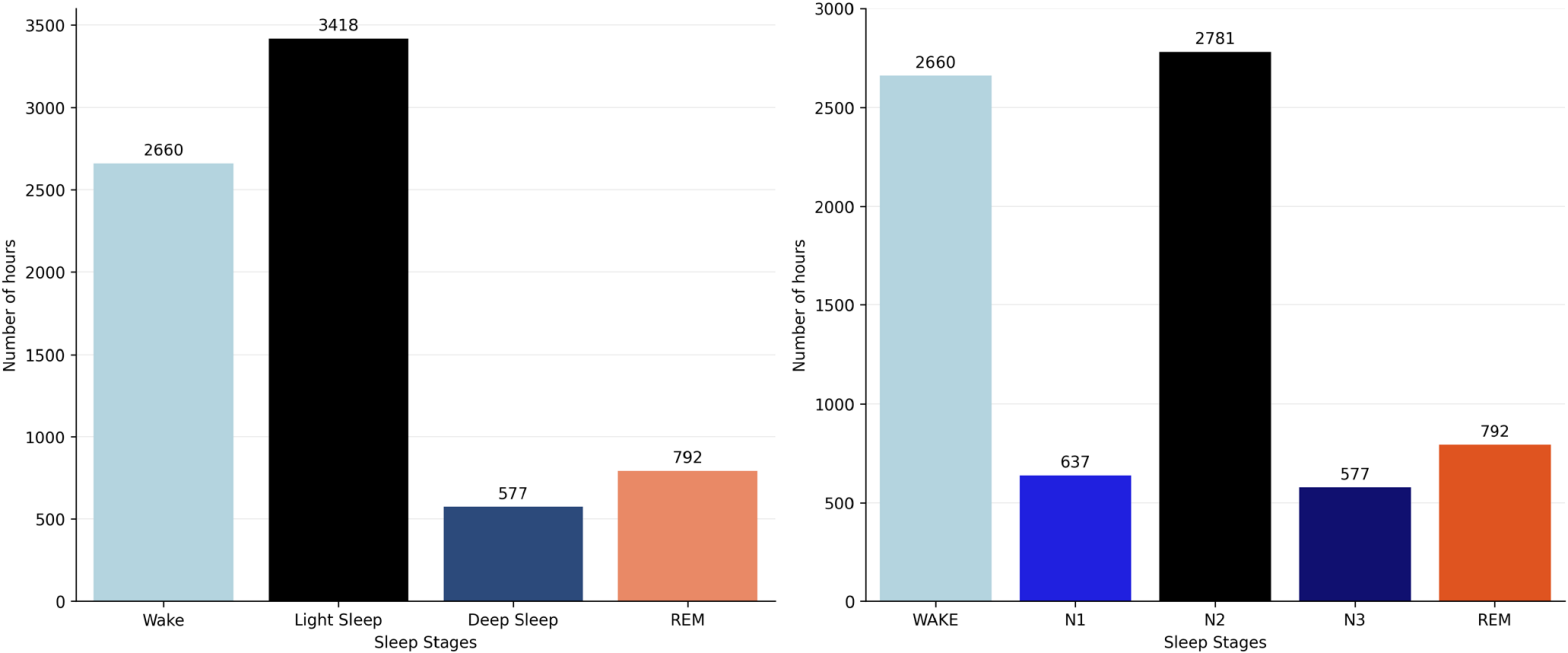
Class distribution in the MESA dataset for five‐stage and four‐stage sleep stage recognition.

The most effective model for classifying five‐ and four‐phase sleep patterns using SHHS data was used for an external evaluation with data not used in the training process. In this case, data from the MESA cohort were used. Therefore, a different dataset assessed the generalisation capabilities with new and unobserved data. The MESA test dataset comprised 931 patients, equivalent to 7448 h of recorded data. The signals in the MESA dataset exhibited a frequency of 1 Hz. The distribution of data from the MESA dataset across the five and four sleep phase classifications is illustrated in Figure 4.

### 2.3 Deep Learning Model

Several deep learning models have shown great results when detecting sleep stages: long‐short‐term memory (LSTM), convolutional neural network (CNN) and fully convolutional neural network (FCNN) (Choo et al. 2023). Despite the promising results, most models found in the scientific literature divide signals into windows of a specific duration (the typical duration is between 30 and 60 s) when classifying sleep stages. It is possible that some sleep stages may not be detected as a result of the signal being divided into windows. Furthermore, the number of data points to be processed may increase if many windows are generated, for instance, by applying overlapping windows. The architecture chosen for this scientific work is a variation of the well‐known architecture known as U‐Net, which is widely used for image segmentation (Ronneberger et al. 2015). The selection of this methodology was motivated by its ability to predict events—in this case, sleep stages—on a per‐second basis. Consequently, the sampling frequency was set to 1 Hz, primarily to reduce the volume of data to be processed and, in turn, the computational cost. This frequency also facilitated subsequent clinical interpretation, as it provided one data point per second. For each subject, the three recorded signals were concatenated into a 3 × 28,800 matrix (three channels, 8 h at 1 Hz). The one‐dimensional multivariate time series was utilised as the input to the U‐Net model. Within this framework, the experimental phase of the study focused on identifying the optimal hyperparameters for a single U‐Net architecture to perform sleep stage classification. In order to replace an ad hoc ‘trial‐and‐error’ strategy with a systematic engineering methodology, a hyperparameter‐optimisation framework was adopted; accordingly, Keras Tuner was employed. It was observed that both the four‐class and five‐class classification models utilised analogous input structures and datasets, with the distinction lying solely in the final classification layer and label mapping. Specifically, the four‐class model incorporated N1 and N2 into a single category termed ‘Light Sleep’, while the five‐class model maintained the distinction between all stages, assigning each stage to its respective class.

#### 2.3.1 Hyperparameter Optimisation, Training and Evaluation of the Model

For the search for the best model to classify five sleep stages and four sleep stages, for both searches, the same search space was defined to be used by the optimisation algorithm through Keras Tuner. The search space for the best hyperparameters can be seen in Table 2.

**TABLE 2:** Search space for the search of the best hyperparameters.

Hyperparameter	Search space
Number of hidden layers (same number for Encoder and decoder)	{2, 3, 4, 5, 6}
Filter map (first layer)	Min value: 64 Max value: 128 Step: 32
Filter step (encoder‐decoder)	Min value: 32 Max value: 256 Step: 32
Max pooling	2
Kernel size	Min value: 3 Max value: 21
Activation layer(s)	Rectified linear unit (ReLU)
Last layer activation	Softmax
Learning rate	{0.01, 0.001}
Transposed convolution layer—stride	2
Loss	CategoricalFocalCrossentropy
Tuner	Hyperband
Optimizer	Adam

It should be noted that the initial division into training (60%), validation (30%) and test (10%) sets was necessary to facilitate hyperparameter optimisation with Keras Tuner, employing the Hyperband algorithm. This tuning process necessitates the use of a dedicated validation set and is incompatible with cross‐validation, given the requirement for each hyperparameter configuration to be evaluated multiple times during the optimisation process. It is important to note that incorporating cross‐validation at this particular stage would have resulted in prohibitive computational costs, which is an important consideration in the context of this paper. Following the identification of the optimal model architecture and hyperparameters via Keras Tuner, an assessment of model stability and robustness was conducted using five‐fold cross‐validation on the combined training and validation sets (with the test set excluded). This step enabled the evaluation of performance variability and the verification that the model was not overfitting to a specific data split. Finally, the optimal model was retrained on the full training and validation set and evaluated on the SHHS2 test set as well as the external MESA dataset.

Keras Tuner is composed of a number of different tuners, including Hyperband, RandomSearch and BayesianOptimization Tuner. The Bayesian optimisation tuner was excluded from further consideration due to its operational characteristics, sometimes analogous to a black box. Hyperband was selected as the optimisation algorithm to identify the optimal hyperparameters. Hyperband employs a random sampling approach to evaluate all possible combinations of hyperparameters, without executing the entire training and evaluation set. Hyperband trains the model for a few epochs with a set of hyperparameter combinations and selects the best candidates based on the results of these few epochs. The process is performed iteratively, with the tuner subsequently running the chosen candidates through the complete training and evaluation set. In this respect, Hyperband is more effective than other tuners, such as RandomSearch, which perform the complete evaluation in each iteration. The goal of the tuner was to achieve the maximum values in terms of the weighted F1 metric, as it was a multiclassification task with unbalanced data.

Since it is a multiclassification task, the *CategoricalFocalCrossentropy* provided by the Keras libraries was used as the loss function. In this way, weights could be assigned to the results based on the data distribution. As there was a significant imbalance in the data, an attempt was made to compensate for this imbalance by doing so. The number of hidden layers in both the encoder and decoder components of the U‐Net architecture is determined by the number of data points; in this study, this number is 28,800 s. This value was selected to align with the dimensional reduction inherent to the U‐Net architecture, where spatial dimensions are progressively halved through downsampling operations in the encoder and subsequently restored in the decoder, ensuring compatibility with the input data structure and optimising feature extraction and reconstruction.

Given that the output of our model can be interpreted as a probability distribution between four and five sleep phases, the final activation layer is configured as a SoftMax layer. This guarantees that the resulting values fall between 0 and 1, allowing them to be used as predicted probabilities.

The number of hidden layers (encoder and decoder) is determined by the number of data points counted, in this case, 28,800 s. Given that the length of the signal is reduced by the max pooling operation for 1D temporal data with a value of 2 at each encoder layer, the input representation is down‐sampled by taking the maximum value in a spatial window of a specific size.

Once the data sets were ready to begin training, the search for the best parameters and hyperparameters for the deep learning model began. The search for the best models lasted around 20 h with a maximum number of epochs of 80.

All stages concerning data loading, analysis, preprocessing and model training were carried out in a local environment. The training, evaluation and testing of the models were carried out using wsl2 on Windows, running a Jupyter notebook in a virtual environment using Python (version 3.10). Python libraries used to create the models and train them were Keras (version 3.1) on Tensorflow (version 2.16.1), NumPy (version 1.26.4) and Scikit‐Learn (version 1.4.1). The data was manipulated, and the models were trained with an i7 processor, 32GB of RAM and a 24GB RTX 3090 GPU.

After finishing the search for the best hyperparameters, the three best models were retrained looking for the optimal epoch. During the search for the best epoch, callbacks with an *EarlyStopping* of 12 and *ReduceLRonPlateau* of 8 were used. In this way, we had the best configuration for the models and the optimal number of epochs. To validate the performance of the model on other sets of patients, a k‐fold cross‐validation was also performed for each model. Once cross‐validation was performed, the models were re‐trained on the entire training and validation set. Finally, the models were tested on the SHHS2 and MESA test set.

## 3 Results

The results obtained for the classification of five sleep stages and for four sleep stages are presented below.

### 3.1 Five Sleep Stage Classification

The optimal epoch after training the best‐performing model was 30. To validate the performance of the model, we applied cross‐validation with *k = 5*. The model obtained a mean categorical accuracy (acc) of 58.30% and an F1‐score (F1) of 60% on the SHHS2 test through cross‐validation. The model obtained the following results for the different folds: 60.26% acc and 63.71% F1 for fold 1, 58.50% acc and 59.92% F1 for fold 2, 53.52% acc and 53.91% F1 for fold 3, 60% acc and 61.80% F1 for fold 4, and 59.22% acc and 60.53% F1 for fold 5.

The results after the training and validation process indicate that the best model with U‐Net architecture for the classification of sleep stages for five stages includes the values for the hyperparameters listed in Table 3.

**TABLE 3:** Best set of hyperparameters for the deep learning model in the five sleep‐stage classification.

Hyperparameter	Best value
Number of hidden layers (same number for encoder and decoder)	2
Filter map (first layer)	64
Filter step (encoder‐decoder)	160
Kernel size	15
Learning rate	0.001

Once cross‐validation was applied, the model was trained on the entire SHHS2 training and validation set and tested with the SHHS2 and MESA test sets. The different results for each of the metrics used can be seen in Tables 4 and 5.

**TABLE 4:** Metric results for the classification of five sleep stages on SHHS2.

	Precision	Recall	F1‐score
WAKE	84	83	84
N1	11	54	19
N2	73	50	60
N3	47	56	51
REM	85	75	79
Categorical accuracy		65	
Cohen's Kappa		0.61	
Macro avg	60	64	59
Weighted avg	73	65	68

**TABLE 5:** Metric results for the classification of five sleep stages on MESA.

	Precision	Recall	F1‐score
Wake	80	79	80
N1	21	47	29
N2	68	47	56
N3	35	49	41
REM	72	63	68
Categorical accuracy		68	
Cohen's Kappa		0.55	
Macro avg	55	57	55
Weighted avg	66	60	62

**FIGURE 5:**
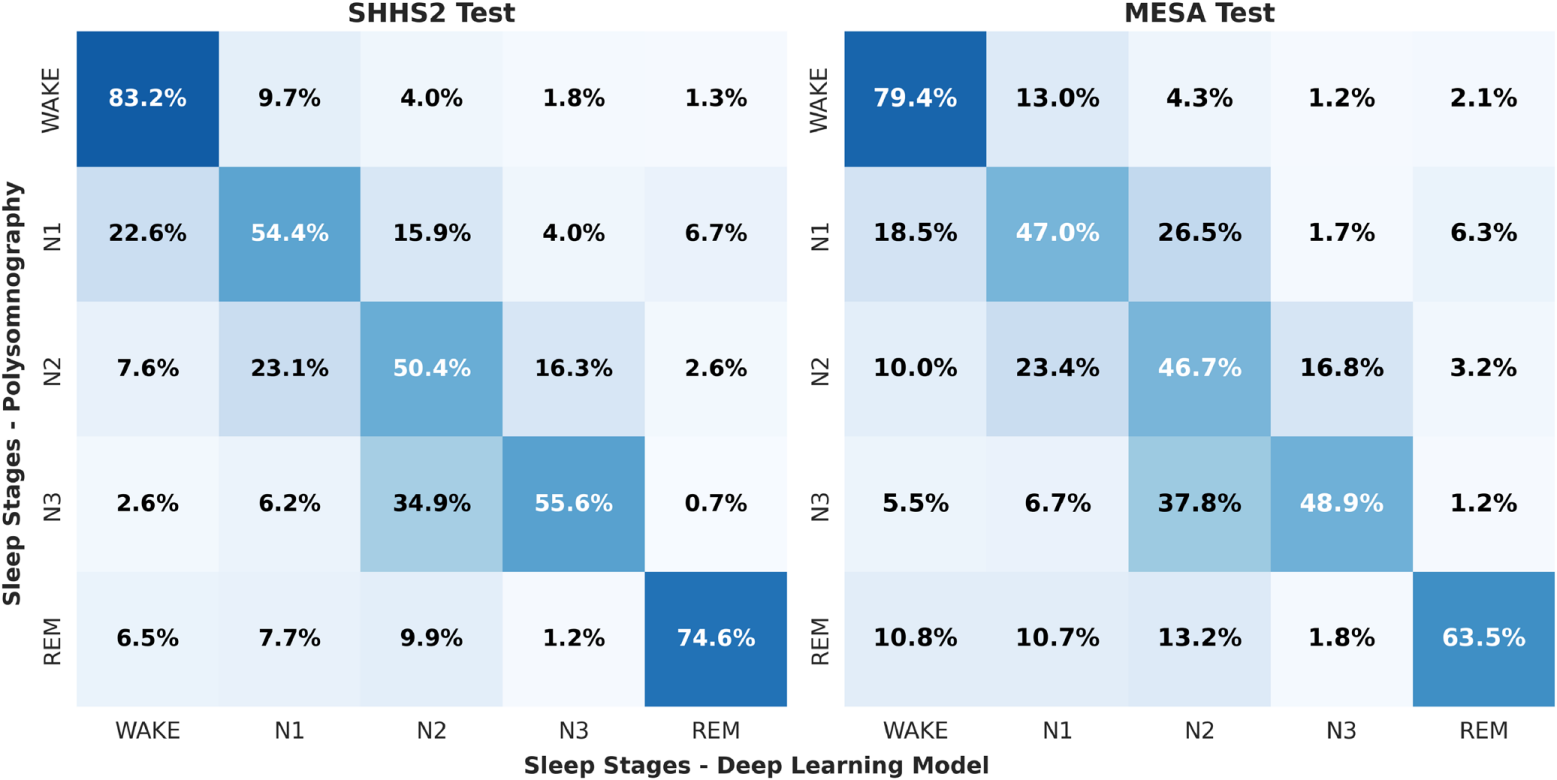
Confusion matrix for the SHHS2 and MESA test sets in the recognition of five sleep phases. The matrices demonstrate the precision of the model in distinguishing between sleep stages (Wake, N1, N2, N3 and REM) as recorded by PSG. Diagonal values (e.g., 83.2% for Wake in SHHS2, 79.4% in Mesa) indicate correct classifications, with higher percentages reflecting better performance. The model demonstrates a high level of accuracy in detecting Wake (83.2%, 79.4%) and REM (74.6%, 63.5%) stages, but exhibits lower precision for N1 (54.4%, 47.0%) and N3 (55.6%, 48.9%), suggesting difficulties in differentiating between lighter and deeper sleep stages.

To more accurately assess the strengths and weaknesses of the model and allow for more in‐depth analysis, the confusion matrix depicting the one‐second event classification for each sleep stage is presented in Figure 5.

### 3.2 Four Sleep Stage Classification

The optimal epoch after training the best model for the recognition of four sleep phases is also 30 epochs. Regarding the recognition of five sleep stages, cross‐validation with *k = 5* was also applied. Mean categorical accuracy of 69.8% and an F1 score of 60% on the SHHS2 cross‐validation test. The model obtained the following results for the different folds: 66.90% acc and 71% F1 for fold 1, 72.73% acc and 73.16% F1 for fold 2, 71.60% acc and 72.72% F1 for fold 3, 67.50% acc and 69% F1 for fold 4 and 70.26% acc and 70.77% F1 for fold 5.

As for the best model for the four‐stage sleep classification, details on the architecture and hyperparameters are shown in Table 6. Following the same steps as for the five‐sleep stage classification model, the results obtained for the four‐sleep stage classification are shown in Tables 7 and 8.

**TABLE 6:** Best set of hyperparameters for the deep learning model in the four sleep‐stage classificaition.

Hyperparameter	Best value
Number of hidden layers (encoder‐decoder)	2
Filter map (first layer)	64
Filter step (encoder‐decoder)	64
Kernel size	13
Learning rate	0.001

**TABLE 7:** Metric results for four sleep stage classification on SHHS2.

	Precision	Recall	F1‐score
Wake	90	76	82
Light sleep	74	61	67
Deep sleep	44	68	53
REM	66	90	76
Categorical accuracy		71	
Cohen's Kappa		0.67	
Macro avg	68	74	70
Weighted avg	74	71	71

**TABLE 8:** Metric results for the four sleep stage classification on MESA.

	Precision	Recall	F1‐score
Wake	90	68	78
Light sleep	72	60	66
Deep sleep	31	61	41
REM	47	85	60
Categorical accuracy		66	
Cohen's Kappa		0.59	
Macro avg	60	69	61
Weighted avg	73	66	68

**FIGURE 6:**
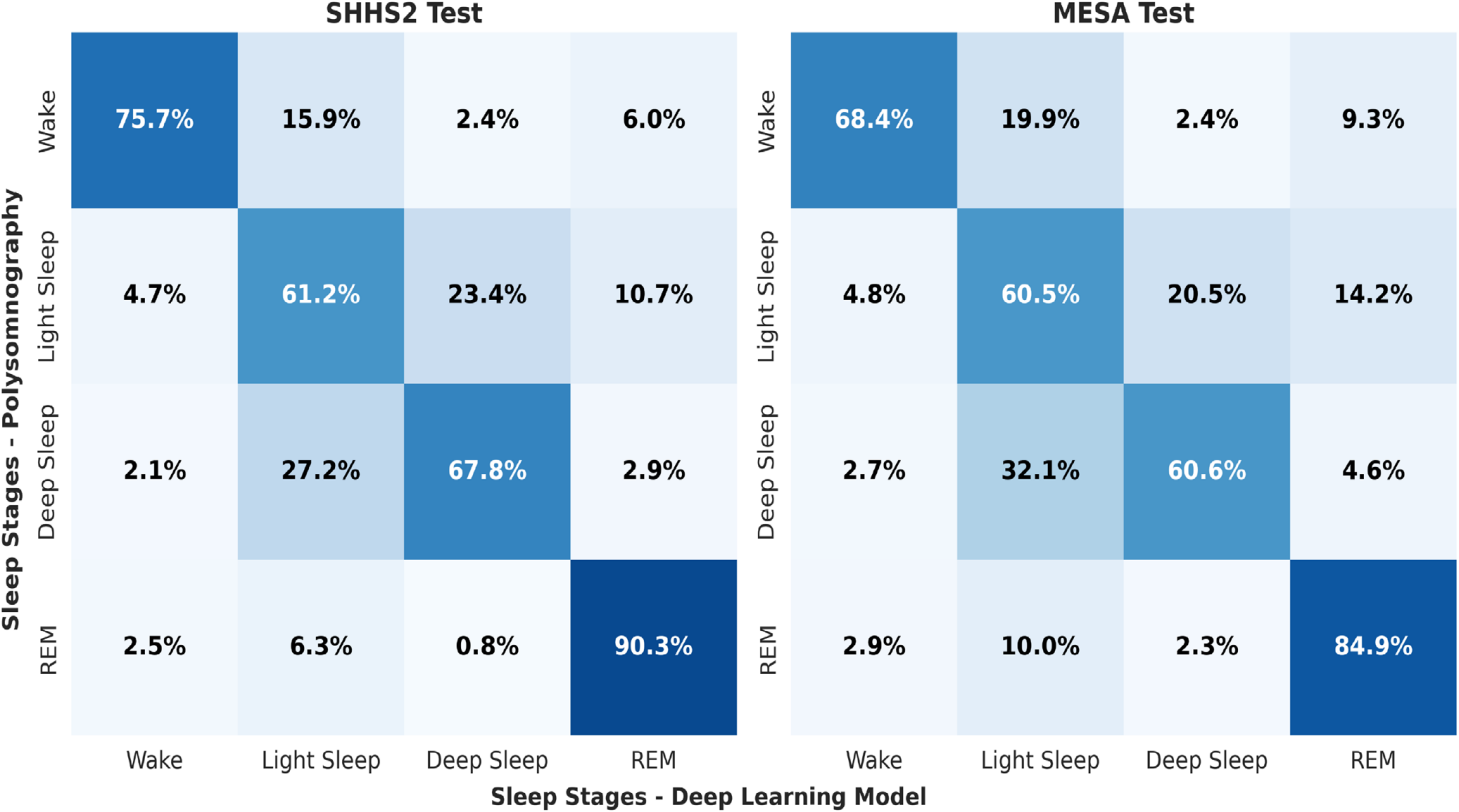
Confusion matrix for SHHS2 and MESA test sets in the recognition of four sleep phases. Sleep stages are categorised into the following: Wake, Light Sleep (N1 + N2), Deep Sleep (N3) and REM. Diagonal values indicate correct classifications, with the model performing well for REM (90.3% SHHS2, 84.9% Mesa) and Wake (75.7%, 68.4%), but showing lower accuracy for Light Sleep (61.2%, 60.5%) due to misclassification with Deep Sleep (e.g., 23.4% in SHHS2). The matrices demonstrate the efficacy in detecting REM sleep, while also highlighting the challenges in differentiating between the stages of light and deep sleep.

As with the five‐stage sleep classification, the confusion matrix, which depicts the one‐second event classification for each sleep stage, is presented in Figure 6.

**FIGURE 7:**
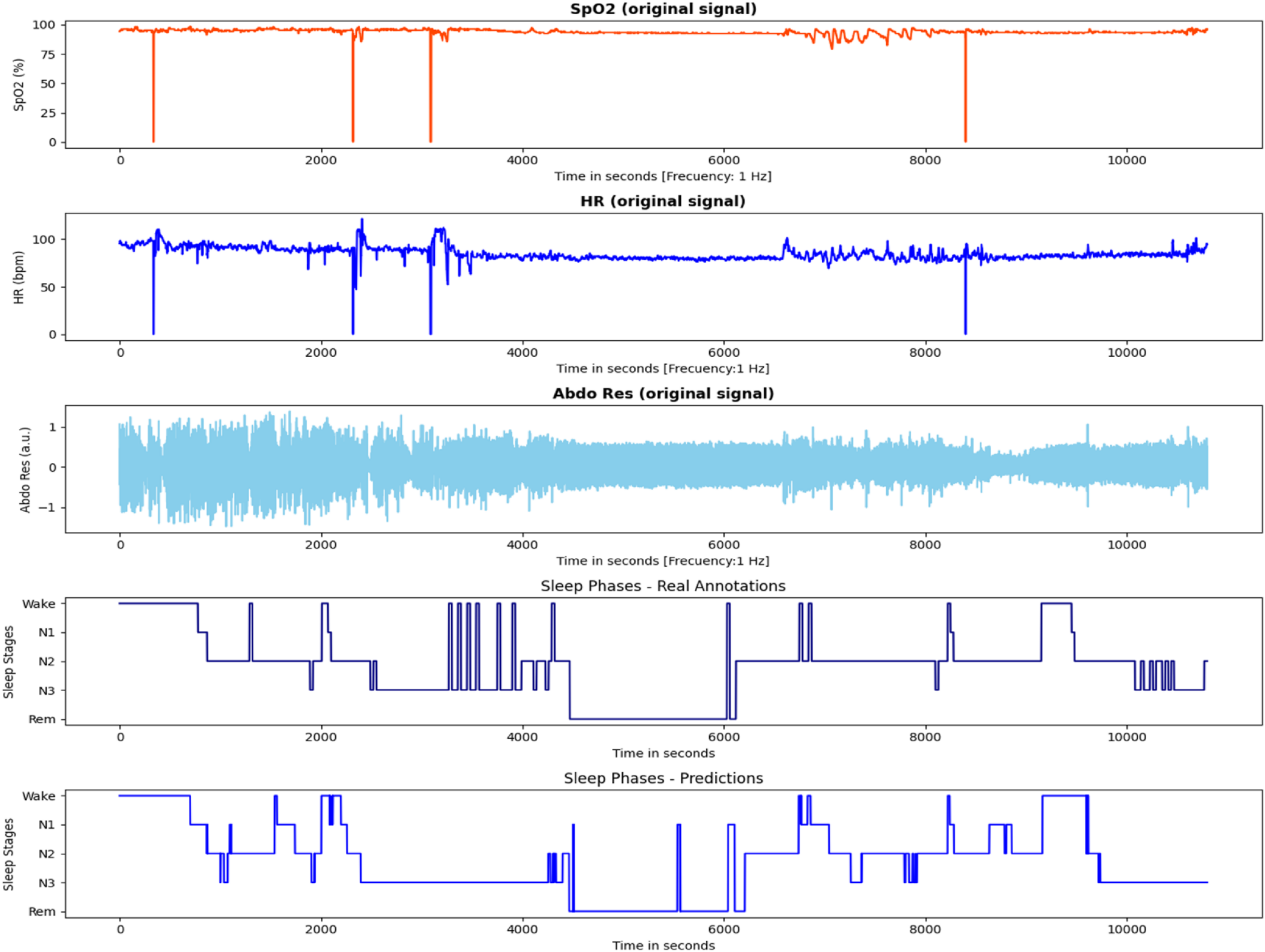
The hypnogram, which depicts five sleep phases, was generated from a set of three signals (SpO_2_, HR and AbdRes) and the predictions made by the deep learning model. Additionally, the figure includes annotations made by the physician.

**FIGURE 8:**
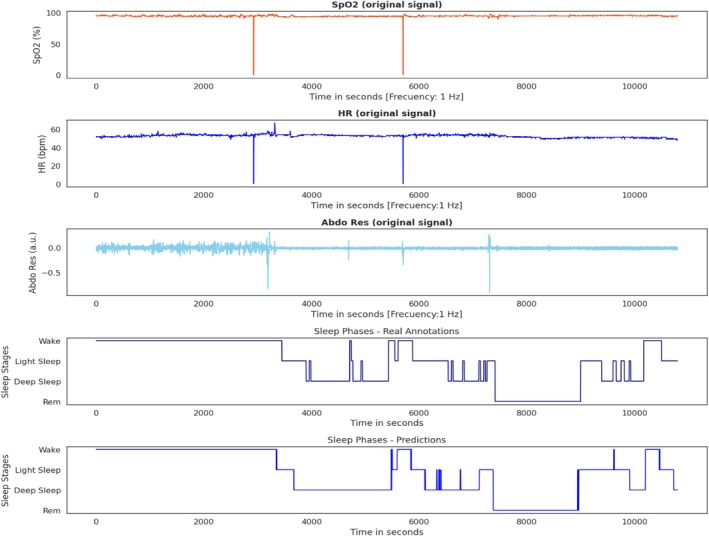
The hypnogram, which depicts four sleep phases, was generated from a set of three signals (SpO_2_, HR and AbdRes) and the predictions made by the deep learning model. Additionally, the figure includes annotations made by the physician.

Finally, after presenting the results for the main metrics, the visualisations of the hypnograms using the best models for the five and four‐sleep stage classification are shown in Figures 7 and 8.

### 3.3 Comparison of Model Performance

As stated previously, one of the objectives of this study is to use a minimum set of signals that can be comfortably acquired from the patient. Consequently, the employment of EEG was dismissed due to the complexity of the measurement setup and the need for an expert assistant. Examining other works that aim to detect sleep phases from a minimal set of signals reveals two distinct categories: those that employ EEG and those that utilise alternative signals to EEG. Models such as DeepSleepNet, AttnSleep and TinySleepNet are among the best performing in terms of sleep stage detection. However, all use raw single‐channel EEG, so a direct comparison with our study is impossible, as they use a very different set of signals. As for U‐Sleep, it uses an arbitrary combination of EEG and EOG channels, so it would not be comparable to our dataset. This poses a significant challenge in directly comparing our work with existing studies, primarily due to the limited set of signals and the distinct preprocessing methods employed.

Taking into account the points above and regarding scientific works employing an alternative set of EEG signals, in Huttunen et al. (2023), they used a single ECG (HR) signal derived from data collected from 50 patients from their acquisition of PSG. This study claims an overall accuracy of 72% for the recognition of four phases of sleep and 66% for five phases of sleep. In Kim et al. (2022), the researchers used a combination of PPG and oxygen saturation and a separate set of PPG, SpO_2_ and nasal pressure. Data for this study were obtained from 933 PSG recordings collected between 2015 and 2017 at the Sleep Disorders Centre, Princess Alexandra Hospital, Brisbane, Australia, at 32 Hz. This work obtained results similar to those obtained here for the recognition of five sleep phases with 69% accuracy and 68% F1 for the first dataset and 70% accuracy and 68% F1 for the second. In Mc Carthy (2021), they obtained an accuracy of 73.9% and a Cohen's kappa of 0.55 for four sleep phases with a set of signals, including respiration, HR and ballistocardiograph (BCG). For the SHHS test, the study obtained an accuracy of 71% and a Cohen's kappa of 0.67. The MESA test yielded an accuracy of 66%, accompanied by a Cohen's kappa of 0.59. In Sridhar et al. (2020), instantaneous HR was utilised from both the SHHS and MESA datasets. This study, which focused on detecting four sleep phases, achieved 77% accuracy and 0.67 Cohen's kappa for SHHS and 80% accuracy and 0.69 Cohen's kappa on MESA.

Despite the fact that the previously exposed works do not use the identical set of signals employed in the present scientific study, the studies mentioned above achieve a good level of performance in terms of the recognition of stages four and five when using a minimal set of signals. However, these studies show significant differences compared to our work. For instance, these studies utilise conventional 30‐s periods to classify sleep stages, whereas our work enables the classification of the entire signal second by second. This capability facilitates the task of analysing the signal and prediction after classification. A notable strength of our work is that, despite reducing the sampling rate to 1 Hz to facilitate data processing and algorithm training, the loss of information is minimal. As illustrated in Figures 5 and 6, the classification results for all classes are uniformly distributed, and there are no significant disparities between the different sleep stages, in contrast to the findings in the studies above, where N3 or deep sleep typically exhibits a low recognition rate. A more detailed discussion of these differences can be found in the next section.

## 4 Discussion

Most studies aiming to detect sleep stages through automatic detection models prioritise achieving high performance metrics, such as accuracy, F1 and recall, over developing reproducible methodologies that can be extended or transferred to other datasets. In contrast, our work emphasises a systematic approach to model development, facilitating replication and evaluation. For example, in Huttunen et al. (2023), data from 50 patients were used, revealing significant disparities in recognition rates, particularly low for Wake, N1 and N3 stages, unlike our study, which achieves more consistent classification across all sleep phases with a larger dataset. Similarly, Kim et al. (2022) developed a U‐Net‐based model for sleep stage detection using alternative datasets with non‐EEG signals, employing 30‐s windows and a dataset split of 80% training, 10% evaluation and 10% testing. Our approach, however, achieves per‐second predictions, yielding comparable or superior results, such as improved recall for N1, despite using fewer data points. Additionally, unlike Kim et al. (2022), which lacks cross‐validation and testing on unseen datasets, our study verifies generalisation power using the MESA dataset. In comparison, Mc Carthy (2021) used 30‐s windows with a 200 Hz sampling rate and tested on a small dataset of 20 individuals, whereas our model demonstrates robustness with higher resolution and a larger test population. Furthermore, Ronneberger et al. (2015) report lower recognition rates for deep sleep compared to other phases, while our work maintains balanced performance across all sleep stages, highlighting the robustness of our methodology. From an engineering perspective, per‐second prediction facilitates high‐resolution sleep stage tracking without the constraints imposed by fixed‐length windows (e.g., 30‐s epochs). This approach reduces the risk of temporal averaging errors and increases flexibility in downstream applications such as event detection or edge device deployment. From a clinical standpoint, this high‐resolution imaging may facilitate direct visualisation of micro‐arousals, brief stage transitions and atypical patterns that may be obscured by conventional 30‐s scoring. Furthermore, it facilitates more precise temporal alignment with other physiological events, thereby supporting richer multimodal analyses. Collectively, these advantages may lay the groundwork for more detailed and clinically informative sleep assessment in the future.

The primary objective of this study was to classify sleep stages using a deep learning model developed through a systematic engineering approach. This work provides a detailed description of the pre‐training, training and validation stages, ensuring clarity in model replication and performance evaluation. Such transparency enables researchers to reproduce and extend the models while verifying their classification strength and generalisation to unseen data, such as the MESA dataset, where performance loss is minimal compared to the SHHS2 test set. A key strength of our approach lies in its use of only three physiological signals sampled at 1 Hz, simplifying data manipulation and analysis for clinical applications. This low data complexity also facilitates model training, with convergence achieved in approximately 30 epochs per model. The results demonstrate balanced performance across sleep stages, with no significant decline in any class, underscoring the model's robustness and potential for practical implementation.

Despite the strengths of the model, certain limitations persist, particularly in the classification of stages N1, N2 and N3, where prediction accuracy remains suboptimal. Specifically, the N1 stage poses the greatest challenge, as evidenced by low F1 scores of 19% on SHHS2 and 29% on MESA, consistent with its known difficulty due to its short duration, transitional nature and underrepresentation in datasets like SHHS2 and MESA. The limited data for N1, compared to Wake and N2, and its physiological complexity contribute to poorer performance relative to REM, which benefits from distinct physiological characteristics. Additionally, the model is constrained to signals no longer than 8 h to reduce computational costs and expedite training. This restriction may limit applicability to longer recordings, although potential solutions, such as padding shorter signals to 8 h or training with extended signals, could address this issue in future work.

The ability to predict sleep stages using non‐EEG signals through deep learning models, as demonstrated in this study, has significant clinical potential. By utilising portable monitors and AI, this approach could reduce reliance on PSG, minimising patient invasiveness by eliminating the need for extensive sensor setups and overnight stays in sleep laboratories. The results indicate a strong correlation between manual sleep scoring and model predictions, with acceptable performance on unseen datasets, suggesting progress towards practical clinical applications. However, the moderate accuracy for the N1, N2 and N3 stages highlights that further improvements are needed to match the precision of PSG. However, this study narrows the gap between automated detection and traditional methods, offering a promising framework for accurately predicting sleep stage durations throughout the patient's sleep cycle, a challenge for many existing models.

## 5 Conclusion

Developing an AI algorithm to detect sleep stages has significant potential to reduce the problems encountered in the clinical setting when using PSG. In this scientific work, we presented the novel development of a deep learning model (specifically a version of U‐Net) to detect sleep stages with a minimal set of signals (SpO_2_, HR and AbdRes). The model for the five sleep stages obtained 65% accuracy and 0.61 Cohen's kappa for the SHHS2 test set and 68% and 0.55 for MESA, respectively. For four sleep stages, the model obtained 71% accuracy and 0.67 Cohen's kappa for the SHHS2 test set and 66% and 0.59 for MESA, respectively. This indicates promising results for the model that have the potential to be improved in the recognition of non‐REM phases, mainly N1. In consideration of the limited set of signals utilised, which can be recorded in a straightforward and accessible manner for the user, the substantiated generalisation of the findings through the utilisation of two independent datasets, the diminished footprint generated during the training process and, most significantly, the capacity to predict the sleep phases for every single second, the research undertaken constitutes a substantial enhancement within the domain of sleep analysis and signifies a tangible advancement in comparison to the prevailing state of the art.

## Author Contributions

**Ángel Serrano Alarcón:** conceptualization, investigation, methodology, data curation, formal analysis, writing – original draft, writing – review and editing. **Maksym Gaiduk:** data curation, conceptualization, writing – review and editing, visualization. **Natividad Martínez Madrid:** conceptualization, resources, supervision, funding acquisition, writing – review and editing. **Juan Antonio Ortega:** investigation, funding acquisition, writing – review and editing. **Ralf Seepold:** resources, supervision, funding acquisition, writing – review and editing.

## Funding

This research was partially funded by the German Federal Ministry for Economic Affairs and Energy, ZiM project ‘Sleep Lab at Home’ (SLaH) grant: ZF4825301AW9, Carl Zeiss Foundation: Project ‘Non‐invasive system for measuring parameters relevant to sleep quality’ (project number: P2019‐03‐003) and ‘Generation of Reliable Synthetic Health Data for Federated Learning in Secure Data Spaces’ Grant PID 2022 141045OB‐C42 funded by MCIN/AEI/10.13039/501100011033.

## Conflicts of Interest

The authors declare no conflicts of interest.

## Data Availability

The data that support the findings of this study are openly available in the Sleep Heart Health Study at https://doi.org/10.25822/ghy8‐ks59, reference number NCT00005275.
